# Research on a Train Safety Driving Method Based on Fusion of an Incremental Clustering Algorithm and Lightweight Shared Convolution

**DOI:** 10.3390/s24154951

**Published:** 2024-07-30

**Authors:** Hongping Wang, Xin Liu, Linsen Song, Yiwen Zhang, Xin Rong, Yitian Wang

**Affiliations:** School of Mechanical and Electrical Engineering, Changchun University of Science and Technology, Changchun 130012, China; 2023200119@mails.cust.edu.cn (X.L.); songlinsen@cust.edu.cn (L.S.); zhyw@cust.edu.cn (Y.Z.); 2018025014@chd.edu.cn (X.R.); 2022100514@mails.cust.edu.cn (Y.W.)

**Keywords:** train safety protection, obstacle detection, semantic segmentation, clustering algorithm, YOLOv8

## Abstract

This paper addresses the challenge of detecting unknown or unforeseen obstacles in railway track transportation, proposing an innovative detection strategy that integrates an incremental clustering algorithm with lightweight segmentation techniques. In the detection phase, the paper innovatively employs the incremental clustering algorithm as a core method, combined with dilation and erosion theories, to expand the boundaries of point cloud clusters, merging adjacent point cloud elements into unified clusters. This method effectively identifies and connects spatially adjacent point cloud clusters while efficiently eliminating noise from target object point clouds, thereby achieving more precise recognition of unknown obstacles on the track. Furthermore, the effective integration of this algorithm with lightweight shared convolutional semantic segmentation algorithms enables accurate localization of obstacles. Experimental results using two combined public datasets demonstrate that the obstacle detection average recall rate of the proposed method reaches 90.3%, significantly enhancing system reliability. These findings indicate that the proposed detection strategy effectively improves the accuracy and real-time performance of obstacle recognition, thereby presenting important practical application value for ensuring the safe operation of railway tracks.

## 1. Introduction

With the accelerated process of urbanization, railway, subway, and light rail systems have become crucial cornerstones of modern urban development. These systems not only facilitate efficient connections between cities, but also play essential roles in maintaining the efficient operation of urban life. In this context, ensuring the safety of railway transport has become a primary concern for various types of railway systems, including metro and light rail. As the number, volume, and speed of trains increase [1], the incidence rate of railway crashes rises, posing threats to passenger safety and significantly impacting railway transport efficiency, leading to substantial resource wastage. The issue of foreign object intrusion in railway transport, characterized by its unpredictability, suddenness, and randomness, makes it difficult for railway personnel or drivers to detect potential obstacles in a timely manner, thereby greatly increasing the risk of railway crashes. Consequently, implementing effective monitoring and control measures for railway areas prone to foreign object intrusion has become an urgent task in railway safety management. In this context, real-time, efficient, and accurate detection of foreign objects on railways has evolved into an important research topic. Numerous researchers have proposed various solutions to this problem. Common methods include using sensors to directly or indirectly detect contact between sensing elements and foreign objects. For instance, leaky coaxial cables and grid sensors are widely used for detecting railway obstacles. While detection methods based on leaky coaxial cables have demonstrated good performance in practical applications [2], grid sensors have proven effective in detecting intruders [3]. However, these contact-based detection methods often occupy considerable space and have limited detection ranges. Therefore, in many cases, non-contact detection methods have become a more ideal choice. For example, detection systems based on ultrasound technology utilize signal processing and complementary sequences for coding [4]. Although they can resist random fluctuations in signal transmission to a certain extent, their detection range remains limited.

In recent years, deep learning technology has made significant advancements in the fields of image recognition and pattern detection. By constructing multi-layered neural network structures, deep learning can automatically learn and extract features from data, and is widely applied across domains such as object detection, speech recognition, and natural language processing. In the realm of visual perception, particularly in pattern recognition algorithms based on sensors like cameras, these technologies have greatly improved detection accuracy and efficiency. For instance, the YOLO (You Only Look Once) series of algorithms [5,6,7] transforms object detection tasks into a single neural network regression problem, achieving real-time and efficient object detection and localization. Furthermore, the successful application of transformer models in natural language processing has inspired new approaches and methodologies in image processing, including exploration in tasks such as image generation and recognition [8]. These advancements not only enhance the performance of traditional pattern detection algorithms, but also drive the application of deep learning in complex scenarios.

Therefore, deep learning-based detection algorithms using sensors such as cameras have shown notable progress. In particular, the Rapid Region-based Convolutional Neural Network (R-CNN) model has demonstrated excellent performance in the detection of leading vehicles in traffic monitoring [8]. For example, He et al. [9] proposed a fast and accurate target detector introducing a new up-sampling parallel structure and context extraction module (CEM) in the R-CNN architecture and obtained excellent results. Wei et al. [10] proposed a new real-time small object detection (RSOD) algorithm based on YOLOv3, which improves the accuracy of small object detection by using shallow layer feature maps, making it suitable for predicting more fine-grained information about location; assigning weights to the output features of FPN achieved good detection results. The successful deployment of this technology has partially addressed issues related to occlusions by foreign objects, while significantly reducing false detection rates and enhancing detection accuracy. However, despite the notable advancements in neural network-based object detection, limitations persist. For instance, these networks typically rely on extensive training data, thereby struggling to effectively predict previously unseen or sudden obstacles such as rockfalls or irregular obstacles like tunnel cables. These challenges restrict the widespread application of deep learning-based object detection technologies in ensuring railway operational safety.

Given these limitations of existing technologies, this study proposes a comprehensive railway obstacle detection system. The system integrates an incremental clustering algorithm and enhanced YOLOv8 semantic segmentation technology to address deficiencies in deep learning-based object detection [10], particularly in handling unknown or sudden sample scenarios. Through this approach, the study effectively identifies any unforeseen obstacles within the track boundary, significantly enhancing the overall performance of the detection system. The incremental clustering algorithm adopted in this paper serves as the backbone of detection, combined with dilation and erosion operations from image processing, to effectively recognize continuous point cloud clusters in a two-dimensional polar coordinate space. Simulating image dilation and erosion operations expands the boundaries of point cloud clusters and merges adjacent point cloud elements into the same cluster, more accurately representing potential obstacles. Furthermore, the study effectively integrates the aforementioned methods with the improved YOLOv8 semantic segmentation algorithm to achieve precise obstacle detection. This comprehensive strategy not only significantly improves the real-time detection and accuracy of obstacles ahead of trains, but also contributes substantially to enhancing train operational safety, providing robust technical support for urban rail transportation safety.

## 2. Related Work

This discussion encompasses the application of obstacle detection and intrusion boundary technologies in railway field environments. Consequently, the related work is divided into two parts. The first part discusses research on the intrusion boundary, focusing on methodologies and approaches to delineate and monitor boundaries susceptible to obstacle intrusion within critical railway areas. The second part introduces algorithms specifically designed for obstacle detection using LiDAR technology. This section explores advancements in processing LiDAR data to identify and classify obstacles such as debris, vegetation encroachment, or structural impediments that could impact railway operations or safety.

### 2.1. Track Transgression Detection

Since the inception of railways, ensuring operational safety has been a paramount concern. Among the challenges faced in railway safety operations is the detection of intrusion boundaries, which constitutes a significant area of research. Early methods for detecting intrusion boundaries in railways involved direct or indirect contact using grid or mesh sensors to acquire obstacle information. These methods are characterized by straightforward data processing and mature technology. While effective to a certain extent in identifying intrusion events, they are associated with complex installation, substantial investment, and limited detection range. Due to the drawbacks of contact-based detection methods, non-contact detection methods have emerged. These methods utilize the propagation characteristics of electromagnetic and acoustic waves to gather obstacle information without physical contact with the object surface. The data obtained from these methods contain abundant information but require complex data processing. Researchers have employed thermal imaging cameras for detecting objects in designated railway areas [7], achieving good results in low-light environments and detecting biological obstacles. However, these methods are unable to detect obstacles at the same temperature as the environment and do not provide depth information. In the railway domain, numerous vision-based object or foreign object detection methods have been developed [11,12]. These methods typically employ artificial intelligence and computer vision techniques for obstacle detection. Among these, vision-based object detection algorithms are one of the most active research areas in computer vision [12,13,14,15]. LiDAR sensors [16,17] provide precise distance information for objects and have been widely used. Guan et al. [18] proposed a lightweight three-stage detection framework for identifying obstacles in a single railroad image consisting of a Coarse Region Proposal (CRP) module, a lightweight Railroad Obstacle Detection Network (RODNet), and a post-processing stage for identifying obstacles in a single railroad image.

Traditional railway inspection methods rely on LiDAR technology to obtain accurate three-dimensional information about the railway and its surrounding environment. Existing studies referenced in this research [19] have employed the RANSAC algorithm to segment point clouds of the ground and railway, demonstrating high accuracy and efficiency in railway scenarios. This method effectively detects obstacles by precisely defining the railway’s region. However, while LiDAR excels in short-range performance, its capability for long-range detection is severely limited, typically covering only 70 to 100 m. Beyond this distance, the quality of point cloud data sharply declines, making it challenging to meet the broader detection range requirements of railway safety.

To overcome this limitation and enhance the long-range capabilities and reliability of railway inspection technology, recent research [20,21,22] has employed deep learning-based object detection networks to detect obstacles that breach intrusion boundaries. This approach has significantly improved detection accuracy and real-time performance. Specifically, the network utilizes D-CSPDarknet as a feature extraction network and optimizes feature fusion through path aggregation and feature pyramid networks, significantly enhancing the model’s capability to detect obstacles at medium to long distances. Additionally, spatial pyramid pooling technology further enhances the network’s adaptability to obstacles of different sizes. However, despite technological breakthroughs, this method still relies on supervised learning, potentially limiting its effectiveness in detecting unknown obstacles. If training data are insufficient to cover all possible scenarios, the model may fail to correctly identify unfamiliar obstacle types. This learning limitation is particularly critical in railway safety applications, where uncommon obstacles may appear in operational scenarios but have not been learned by the model, thus impacting train operational safety [23]. In summary, while current methods have made progress in railway track segmentation and intrusion boundary detection, challenges remain in detecting unknown obstacles and achieving long-range detection. This underscores the urgent need in railway safety research to develop new technologies capable of adapting to unknown environments and enhancing overall detection capabilities.

### 2.2. 3D Obstacle Detection

The obstacle detection algorithms for point clouds mainly include several clustering and deep learning algorithms. The principle of clustering analysis is to group samples with high similarity into the same class, maximizing the similarity within each class while minimizing the similarity between different classes, to reveal the actual distribution of the data. The DBSCAN algorithm is a density-based clustering algorithm that can rapidly cluster point cloud data of any shape [17]. For each object in a cluster, the number of data objects within a given radius must exceed a specified value. In other words, the neighborhood density must surpass the density threshold. The algorithm identifies any object in the dataset and finds objects satisfying the given radius and density threshold to form clusters within the dataset. This method consumes significant memory when handling large quantities of point clouds.

The k-means clustering algorithm ensures accuracy and efficiency of clustering results with massive datasets [24]. It requires input of sample data and the number of clusters equal to K. Initially, K objects from the samples are chosen as initial clusters, their centroid positions computed, and distances from all points in the sample to each centroid calculated. Points are then reassigned based on minimum distances; centroid positions and distances are recalculated, and points are reassigned iteratively until centroids no longer change. This method requires manual input of K and may not be suitable for complex obstacle detection environments.

Deep learning-based clustering analysis integrates semantic information from point clouds for clustering, enhancing target localization accuracy, albeit requiring substantial data support [25]. For example, Sun et al. [26] proposed a road obstacle detection method based on highly analyzed extracted 3D candidate point clouds, applying a variant of RANSAC to reduce point cloud matching errors in 3D reconstruction. Insufficient data may hinder achieving desired outcomes. Hence, this paper proposes an incremental clustering algorithm. This algorithm aims not only for high detection accuracy, but also emphasizes consistent noise handling and precise detection at longer distances, reducing instances of false positives and negatives. Moreover, the algorithm particularly stresses efficiency in real-time data processing to ensure rapid response and decision support capabilities in dynamic environments.

## 3. Method

### 3.1. System Architecture

**Figure 1:**
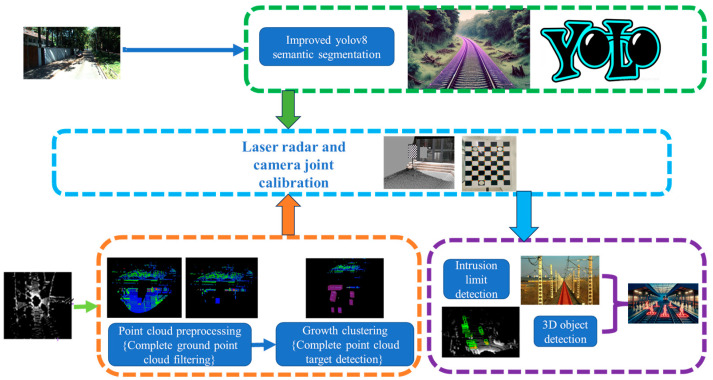
Construction of obstacle detection system.

In this study, we propose an advanced obstacle detection solution that integrates the incremental clustering algorithm with lightweight shared convolutional segmentation technology. This approach aims to address the rapid and accurate identification of obstacles in railway track transportation, as illustrated in Figure 1. Firstly, we introduce an enhanced detection region segmentation scheme based on an improved RANSAC algorithm. The enhanced RANSAC algorithm employs line pass filters to extract track areas and conducts an initial screening on the raw point cloud to gather a comprehensive set of track points. A phased RANSAC algorithm is utilized for precise ground fitting, integrating dynamic thresholding techniques to effectively segment and process ground point cloud data across different distance layers. This method not only enhances the efficiency of point cloud data processing, but also ensures precise segmentation of track gradients, which is particularly crucial for long-distance detection. This process significantly improves the accuracy and reliability of obstacle detection.

To further enhance the detection system’s performance, we process the laser radar point cloud data using the incremental clustering algorithm. This algorithm effectively removes noise and identifies spatially adjacent point cloud clusters, providing a more precise determination of unknown or emergent obstacles. Additionally, the improved YOLOv8 semantic segmentation algorithm incorporates a proprietary lightweight shared convolutional detection head (LSCD), optimizing the model’s computational efficiency and real-time processing capability. This innovative technological integration not only enhances the accuracy of obstacle detection, but also meets real-time requirements, thereby significantly improving railway transportation safety.

### 3.2. Ground Point Cloud Filtering

To further enhance the performance of the detection system, particularly in handling complex railway environments, we propose a detection area segmentation scheme based on an improved RANSAC algorithm. Describing railway scenes from large datasets composed of 3D points requires meticulous analysis, as the complexity of the algorithm increases with the number of points, leading to longer computation times and potential degradation in detection accuracy due to noise interference. Effective boundary area segmentation can significantly improve detection speed and accuracy. Innovatively building upon the RANSAC algorithm, we optimized the segmentation scheme for detecting areas. Specifically, by employing a line filter to extract the track area and conducting preliminary filtering of the raw point cloud, we aim to capture as many points on the track as possible. Central to this process is the phased application of the RANSAC algorithm to precisely fit the ground surface, accommodating differences in the quality of ground point cloud data across various distances. We introduced dynamic thresholding techniques to effectively segment and process ground point cloud data at different distance levels. Through this approach, we not only enhance the efficiency of point cloud data processing, but also achieve precise segmentation, especially addressing the impacts of track slopes in long-distance detection, thereby ensuring the accuracy and reliability of obstacle detection.

#### 3.2.1. Preprocessing of Point Cloud Data

The preprocessing formulas for point cloud data are shown in Equations (1) and (2).

(1) $$P_{filtered}=\{p\in P|\neg isInvalid(p)\}$$

(2) $$\begin{array}{l} isInvalid(p)=(isNaN(p\cdot x)\vee isNaN(p\cdot y)\vee \\ isNaN(p\cdot z))\vee ((\left|p\cdot x\right|<\delta )\wedge (\left|p\cdot y\right|<\delta )\wedge (\left|p\cdot z\right|<\delta )) \end{array}$$

where P represents the original point cloud set and $P_{filtered}$ represents the filtered point cloud set. The function isInvalid(p) returns a Boolean value indicating whether point P is invalid. Here, isNAN() checks if the coordinates are NaN values, and δ is a small threshold used to determine if a point is too close to the origin. If any coordinate of the point is NaN or if all coordinates are close to the origin, then the point is considered invalid.

#### 3.2.2. Spatial Segmentation of Point Cloud

After processing, point cloud data are typically spatially segmented into multiple sectors, which can be represented using polar coordinates:

(3) $$S_{i}=\{p\in P_{filtered}|\theta_{i}\leq a\tan2(p\cdot y,p\cdot x)<\theta_{i+1}\}$$

Here, $S_{i}$ represents the point cloud set in the i sector, and $\theta_{i}$ and $\theta_{i+1}$ are the angular limits of the sector division.

#### 3.2.3. Selection and Sorting of Ground Points

In each sector, points in the point cloud are sorted based on their vertical height (z-value), and points below a specific threshold are selected as ground points, as shown in Equation (4).

(4) $$G_{i}=\{p\in S_{i}|p\cdot z<z_{thres}\}$$

$G_{i}$ is the set of ground points in the i sector, and $z_{thres}$ is the height threshold for selecting ground points.

#### 3.2.4. Fitting Ground Plane

The ground plane fitting is performed using the RANSAC algorithm, and the ground plane can be represented by the equation ax+by+cz+d=0, as shown in the final Formula (5).

(5) $$\min{\sum_{p\in G}\left|ax_{p}+by_{p}+cz_{p}+d\right|}^{2}$$

Here, G is the collection of ground points from all sectors, and $\left(x_{p},y_{p},z_{p}\right)$ represents the coordinates of point P.

#### 3.2.5. Separation of Ground and Non-Ground Points

Based on the fitted ground model, ground points can be separated from non-ground points, as shown in Formula (6).

(6) $$NG=\{p\in P_{filtered}\|ax_{p}+by_{p}+cz_{p}+d|>\varepsilon \}$$

In the formula, NG represents the set of non-ground points, and ϵ is the threshold used to differentiate between ground and non-ground points. Considering the influence of slopes, segmented fitting is used. For long-distance detection, a dynamic threshold RANSAC algorithm is employed to fit the ground model segment by segment to eliminate the influence of slopes, as shown in Formula (7).

(7) $$\forall d_{i}\in D,apply\;RANSAC\;\mathrm{on}\;\{p\in P_{filtered}|d_{i}\leq dis\tan ce(p)<d_{i+1}\}$$

**Figure 2:**
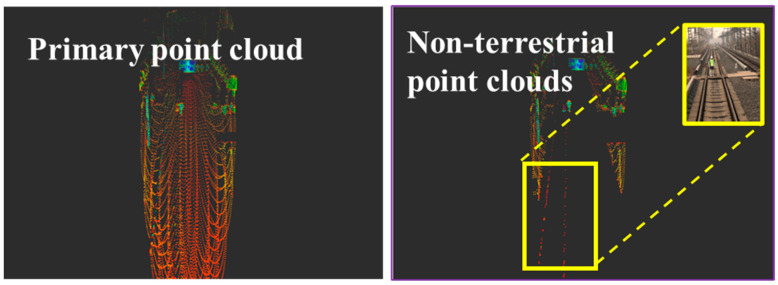
Ground point cloud segmentation diagram.

D represents the set of segmented distances, where distance(p) calculates the distance from point p to a reference point (such as a LiDAR device), to accomplish the segmentation task of ground and non-ground point clouds. As shown in Figure 2, it illustrates the actual segmentation result of ground from LiDAR.

### 3.3. Research on Train Obstacle Detection Algorithm

In the research of LiDAR-based object detection, particularly in the domain of obstacle detection, point cloud clustering algorithms have rapidly advanced due to their capability to provide precise three-dimensional spatial information. This algorithmic approach holds significant advantages in applications such as autonomous driving and railway transportation because of its accuracy in locating and identifying various obstacles. Unlike deep learning methods, a key advantage of point cloud clustering is its independence from extensive training data, enabling effective handling of unknown or unexpected types of obstacles, which is crucial for railway safety.

However, point cloud clustering faces a challenge: it may indiscriminately cluster all scanned objects, leading to over-detection of obstacles and occasional misidentification of noise or objects outside the boundary. In railway transportation, such false positives are unacceptable as ensuring safe train operations is paramount. To address this issue, this study integrates shared convolutional semantic methods to determine whether clustered obstacles genuinely intrude into safe operational boundaries. Semantic segmentation methods adeptly learn and understand complex railway environment features, significantly enhancing detection accuracy while reducing false alarm and omission rates. The combined application of point cloud clustering and semantic segmentation methods notably enhances the safety of train operations. Point cloud clustering provides precise obstacle localization, while semantic segmentation further discriminates these obstacles with finesse. This synergistic strategy optimally leverages the strengths of both technologies, offering a robust and reliable obstacle detection solution for railway transportation systems. Specifically, in the context of point cloud obstacle detection, we propose a growing clustering approach inspired by morphological operations commonly used in image processing, such as dilation and erosion algorithms. In image processing, dilation expands object boundaries, while erosion contracts them. These algorithms are typically employed for noise removal, element separation, and connection of disjointed elements. In point cloud data processing, this concept is applied to three-dimensional point cloud clustering algorithms, aiming to identify continuous clusters of points within a two-dimensional polar coordinate space. Simulating erosion operations helps eliminate surrounding point cloud noise, reducing the misidentification of obstacles and enhancing detection accuracy. Leveraging dilation extends the boundaries of point cloud clusters, merging neighboring point cloud elements into the same cluster, thereby more accurately identifying potential physical objects or obstacles. Specifically, this clustering process initially labels initial point cloud clusters in a polar coordinate grid and progressively expands these clusters, incorporating surrounding neighboring point cloud elements into their respective clusters. This process not only enhances clustering accuracy but also ensures a better representation of actual objects in physical space, particularly in critical applications like obstacle detection. Moreover, this algorithm avoids the need for extensive training data required by traditional deep learning methods, offering greater flexibility and adaptability for handling unknown or unforeseen scenarios, which is crucial for real-time analysis and decision-making. Through this approach, we present an innovative detection solution for safe railway operations.

#### 3.3.1. Creation of Polar Coordinate Grid

Firstly, initialize parameters to define the polar coordinate system for constructing a grid model: polar angle resolution Δθ, distance resolution Δr, maximum detection range $R_{\max}$, and height threshold $Z_{\max}$. Map non-ground point clouds into the polar coordinate grid: for each point p(x,y,z) in the point cloud, calculate its polar coordinate (θ,r).

(8) $$\theta =\arctan2(y,x),r=\sqrt{x^{2}+y^{2}}$$

Map point P to grid $G_{ij}$.

(9) $$G_{iy}=\{p|i\Delta \theta \leq \theta <(i+1)\Delta \theta ,j\Delta r\leq r<(j+1)\Delta r\}$$

#### 3.3.2. The Erosion and Dilation Processes in Agglomerative Clustering Algorithms

For each cell $G_{ij}$ in the polar coordinate grid, initialize a clustering label $Label_{ij}$. During the clustering process, using the erosion and dilation concept from 2D image processing, first define a kernel matrix K. Here, a 3 × 3 matrix is chosen as the kernel matrix.

(10) $$K=\left[\begin{array}{ccc} 1 & 1 & 1\\ 1 & 1 & 1\\ 1 & 1 & 1 \end{array}\right]$$

In this matrix, the value 1 represents neighboring cells considered in the erosion and dilation operations. For each grid cell $G_{ij}$, apply the dilation operation.

(11) $$DilatedCluster_{ij}=\cup_{\left(m,n)\in N(i,j,K\right)}G_{mn}$$

Here, N(i,j,K) represents the set of neighboring grid coordinates around $G_{ij}$ using K as the kernel. Specifically, for the center point $G_{ij}$, its neighboring units are determined by the kernel K, and the point clouds from all neighboring units are merged into the $DilatedCluster_{ij}$ cluster. Based on the clustering result after erosion and dilation, merge clusters of point clouds that are spatially close to each other.

(12) $$MergedCluster_{k}=\cup_{DilatedCluster_{ij}\in AdjacentClusters}DilatedCluster_{ij}$$

Here, AdjacentClusters represents spatially adjacent erosion and dilation cluster groups. The clustering results in polar coordinates are then transformed back to Cartesian coordinates: for each point in the cluster, perform the coordinate transformation using the formula as shown in (13).

(13) $$p^{\prime }(x,y,z)=(r\cos\theta ,r\sin\theta ,z)$$

**Figure 3:**
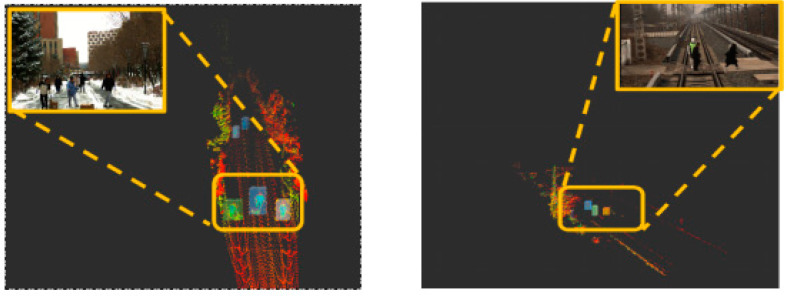
Point cloud clustering diagram.

The proposed incremental clustering algorithm is capable of handling large volumes of point cloud data in real-time, which is crucial for ensuring safe train operations. In terms of accuracy, the algorithm can effectively identify potential obstacles ahead of the train, thereby significantly enhancing the safety performance of train operations. Furthermore, unlike deep learning methods that require extensive training data, this algorithm can detect various obstacles without the need for pre-training. This feature allows the algorithm to demonstrate high adaptability and flexibility in dealing with unknown or unexpected obstacles that may occur in railway traffic scenarios (Figure 3). 

### 3.4. Strategies for Detecting Obstacles in Rail Transportation

In railway transportation systems, timely detection of obstacles is crucial to ensure the safe operation of trains. This section proposes a novel strategy based on enhanced YOLOv8 semantic segmentation technology aimed at efficiently and accurately identifying potential obstacles on the track. This strategy integrates data from both LiDAR (Light Detection and Ranging) and cameras, achieving precise mapping between three-dimensional space and two-dimensional images through joint calibration techniques. This enhancement enhances the accuracy and reliability of obstacle detection across multiple dimensions. Initially, leveraging the precise physical positions of obstacles obtained earlier, the joint calibration matrix between LiDAR and camera data is used to map the corner points of these 3D bounding boxes onto the corresponding 2D image plane. This step is crucial as it integrates the results from 3D detection with predictions from 2D image data, providing a multidimensional perspective for obstacle detection, thereby enhancing the safety of train operations. This section focuses on improving YOLOv8 semantic segmentation technology at the 2D image level. We introduce an adaptive feature selection module in the final layer of the backbone to capture global spatial information of the track images, facilitating region segmentation. Additionally, a custom lightweight shared convolution detection head is integrated into the segment layer to reduce model computational load while ensuring accuracy in identifying railway areas in the images. Through this enhanced algorithm for safe train operation, the safety of train operations is further enhanced.

#### 3.4.1. YOLOv8s-seg Network Model

With the continuous advancements in deep learning technology, YOLOv8 has gained significant popularity across various application scenarios due to its outstanding precision and efficiency. The core of this algorithm lies in its meticulously designed network structure, comprising three key components. Firstly, the backbone utilizes the enhanced residual structure C2F module to effectively extract deep image features, enrich gradient flow information, optimize the training process, and adjust channel numbers for different scales, thereby enhancing the model’s flexibility and adaptability. Secondly, the neck section combines the C2F module with up-sampling modules to achieve multi-scale feature fusion and enhancement, thereby improving detection accuracy and robustness. Finally, the head section adopts a decoupled head structure that separates classification from detection heads, freeing itself from traditional anchor box constraints, simplifying the model structure, and enhancing the detection capability of objects at multiple scales, allowing YOLOv8 to perform exceptionally well in complex scenes.

**Figure 4:**
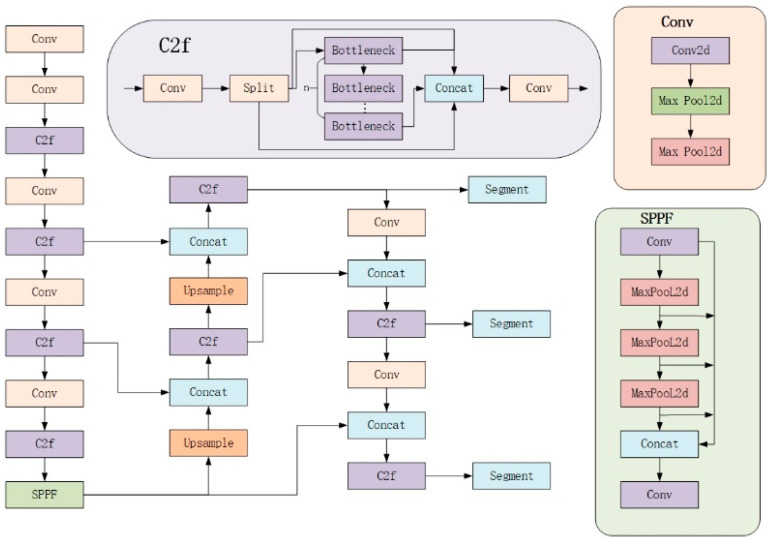
YOLOv8-seg model structure.

Therefore, to meet the high demands for accuracy and real-time performance in railway track obstacle detection, this paper proposes an improved semantic segmentation model based on YOLOv8. YOLOv8-seg is the semantic segmentation model derived from YOLOv8, with its network structure illustrated in Figure 4.

YOLOv8-seg utilizes Darknet as its core backbone network, built upon a series of meticulously designed convolutional layers and residual blocks. These network layers are responsible for accurately extracting crucial features from images and effectively passing them to the subsequent segmentation head for processing. Following the backbone network, YOLOv8-seg integrates a segmentation head dedicated to generating semantic segmentation predictions. This head consists of a series of convolutional and up-sampling layers whose primary task is to restore the feature map size to match that of the original input image, thereby producing pixel-level semantic segmentation results. To further enhance the accuracy and detail representation capability of semantic segmentation, YOLOv8-seg introduces a feature fusion module. Innovatively, this module deeply integrates feature maps from both object detection and semantic segmentation tasks, leveraging the combined information to achieve higher accuracy in semantic segmentation tasks. Ultimately, YOLOv8-seg outputs a semantic segmentation result map that exactly matches the size of the input image. In this result map, each pixel is precisely assigned a class label, clearly identifying the object category or background to which the pixel belongs. In contrast to traditional object detection models, the semantic segmentation model of YOLOv8-seg features unique prototype mask branches and mask coefficients in its head structure. These components work together to generate refined semantic masks, thereby achieving precise segmentation of different semantics. This approach, initially proposed by the YOLACT model and further optimized and applied in YOLOv8-seg, enhances the model’s capability in semantic segmentation tasks.

YOLOv8-seg provides five different model variants, namely, YOLOv8n-seg, YOLOv8s-seg, YOLOv8m-seg, YOLOv8l-seg, and YOLOv8x-seg. According to performance metrics released by Ultralytics in Table 1, these models demonstrate excellent performance on the COCO2017 dataset. Taking into account both model size and performance, this study selects YOLOv8s-seg as the base model, aiming to achieve higher accuracy and efficiency in subsequent research endeavors.

#### 3.4.2. Improving the YOLOv8s-seg Network Model 

**Figure 5:**
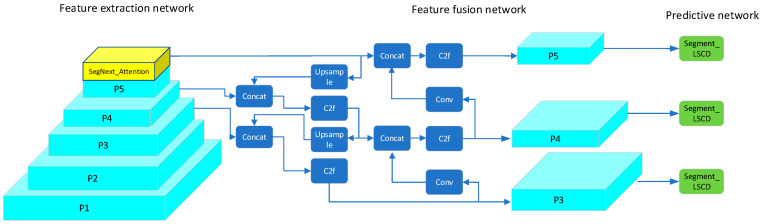
Improved YOLOv8-seg network architecture diagram.

In addressing the segmentation challenges of unknown or emergent obstacles in railway track traffic, models used for railway track segmentation must meet real-time requirements while ensuring detection accuracy. Therefore, this study proposes two main improvements to the YOLOv8s-seg algorithm. Firstly, a self-adaptive feature selection module is integrated into the last layer of the backbone. This module aims to capture global spatial information from railway track images to facilitate region segmentation. Secondly, a self-developed lightweight shared convolution detection head is incorporated into the segment layer. This addition reduces model computational complexity while maintaining accuracy. Figure 5 illustrates the modified network architecture of YOLOv8-seg proposed in this study.

#### 3.4.3. Shared Convolutional Layer Module

The core concept of convolutional neural networks (CNNs) lies in local connectivity and parameter sharing. In convolution operations, parameter sharing is achieved through the use of filters, commonly referred to as weights, which are applied consistently across different spatial locations of the input data. When an image is input into a CNN, these filters scan the image, performing convolutional operations that involve computing the dot product between the filter weights and the local regions of the input. This process allows the network to detect spatial hierarchies of features, such as edges, textures, and more complex patterns, at different levels of abstraction. The advantage of weight sharing is that it significantly reduces the number of parameters in the network, making the model more efficient and less prone to overfitting, particularly when dealing with large-scale image data. Furthermore, local connectivity ensures that the network can effectively capture spatial dependencies within the data, which is critical for tasks such as image recognition, object detection, and semantic segmentation. By leveraging these properties, CNNs have become a fundamental tool in the field of deep learning, enabling the development of highly accurate and computationally efficient models for various computer vision applications.

**Figure 6:**
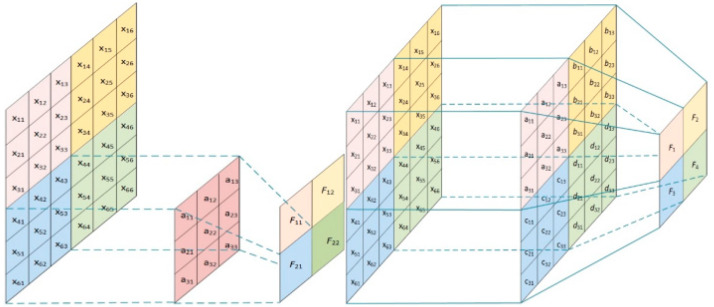
Weight sharing process.

To further optimize convolutional neural networks (CNNs) by reducing their storage requirements and computational burden, this paper proposes an innovative approach: cyclic weight sharing of convolutional layer parameters in the detection layer network. This method effectively reduces redundant parameters within the network, enhancing model efficiency while maintaining strong feature learning and representation capabilities. By implementing cyclic weight sharing of convolutional layer parameters, significant compression of the CNN can be achieved without sacrificing model performance, making it more suitable for resource-constrained environments and real-time applications. Specifically, as illustrated in Figure 6, consider a 3 × 3 convolutional kernel example sliding over an image to extract features. If the input image has k channels, the total number of parameters for this convolutional kernel would be 3 × 3 × k. Without parameter sharing, as depicted in Figure 5, each convolution operation at every position would use independent parameters, resulting in a total of W(width) × H(height) × K(kernel) parameters. For instance, using an input image size of 192 × 192 and removing parameter sharing from the first layer’s 3 × 3 × 32 convolutional kernel, the parameter count would increase to 192 × 192 × 32, which is approximately 4096 times more than the original 288 parameters. Such a large parameter scale not only excessively increases the model’s size, but also escalates the consumption of computational resources and storage space. Based on the above discussion, this paper proposes an optimization strategy within the custom lightweight detection head layer, specifically targeting weight sharing in convolutional layers for classification and regression operations.

#### 3.4.4. Introducing a Self-Developed Lightweight Detection Head

**Figure 7:**
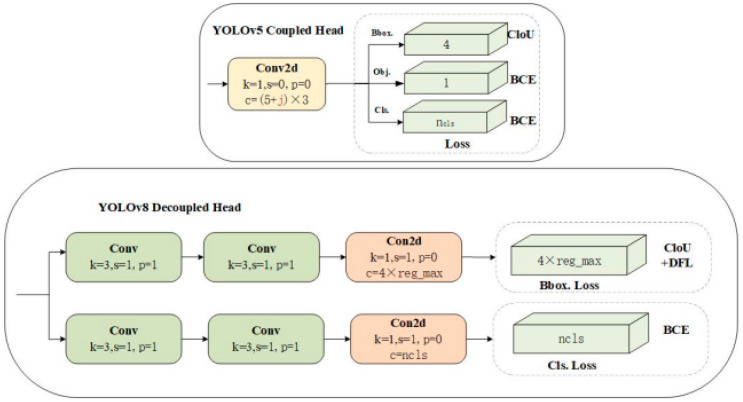
Comparison of detection heads between YOLOv5 and YOLOv8.

YOLOv8 shows the most significant changes in its head section compared to YOLOv5, as illustrated in Figure 7.

From the diagrams, it is evident that YOLOv8 has made significant innovations in its head structure compared to YOLOv5. YOLOv5 originally employed an anchor-based coupled head, which has been transformed into an anchor-free decoupled head design in YOLOv8. This transformation signifies the complete separation of classification and detection heads, discarding the traditional objectness branch in favor of decoupled classification and regression branches that incorporate Distribution Focal Loss (DFL). Each branch undergoes a meticulously designed network structure, including Conv modules with two 3 × 3 convolutional kernels and a Conv2d module with a 1 × 1 convolutional kernel. While this design enhances the model’s capacity for feature extraction and representation, it concurrently increases the computational complexity and parameter count of the model.

**Figure 8:**
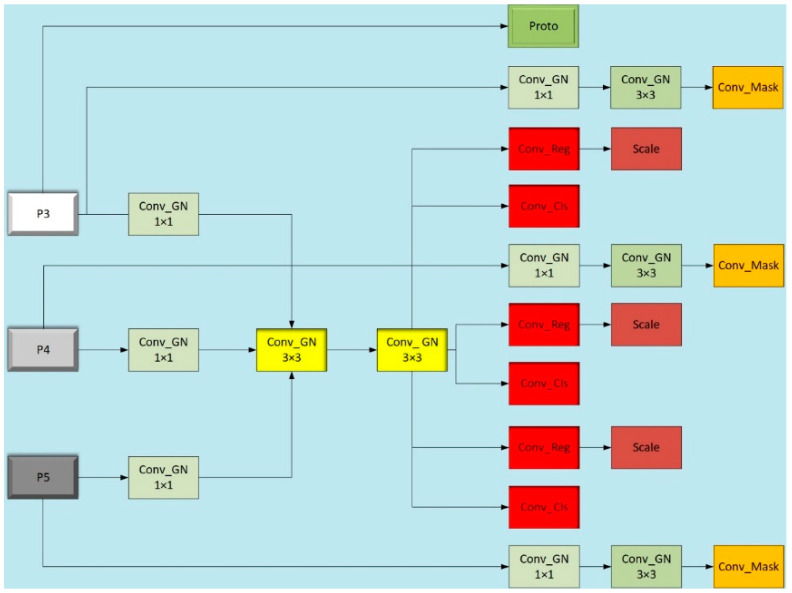
Improvement of the segment layer structure.

To address the randomness and unpredictability of obstacles in railway track traffic, it is crucial to balance model accuracy and efficiency to meet real-time detection requirements. Therefore, this paper proposes an innovative solution: integrating a custom lightweight shared convolutional detection head (LSCD) into the segment structure of YOLOv8s-seg, replacing the original detection head. The design of this LSCD structure aims to reduce the computational burden of the model while maintaining its robust feature learning and representation capabilities. Through this improvement, the new segment structure not only inherits the high precision of YOLOv8, but also significantly reduces computational complexity and parameter count, thereby enhancing the real-time performance of the model. Figure 8 illustrates the segment structure after integrating the LSCD, demonstrating visually how this innovative design optimizes and improves the model structure.

In the YOLOv8-seg model, the segment layer incorporates a crucial component known as the Proto layer, which plays a pivotal role as the prototype mask branch. It is responsible for generating mask coefficients and semantic masks to achieve precise segmentation of different instances in the image. To extract effective features, the network initially passes the feature map through a 1 × 1 convolution module with group normalization (Conv_GN), enhancing feature representation capability while reducing internal covariate shift. Subsequently, the feature map undergoes two 3 × 3 shared convolutional layers with group normalization. These shared convolutional layers not only reduce model parameters and computational load, but also ensure stability and accuracy through their group normalization structure. Following the shared convolutional processing, the feature map is separately processed by Conv_Reg and Conv_Cls convolutional modules for bounding box regression and class prediction. It’s noteworthy that Conv_Reg and Conv_Cls also adopt a shared convolutional design, further enhancing model efficiency. Simultaneously using shared convolutions, the model introduces a scale layer to scale features, addressing scale differences in targets detected by different detection heads, and thus ensuring accurate detection across various object scales.

In the YOLOv8-seg model, another critical component is the Conv_Mask module, which learns and predicts masks for target objects from input features, enabling precise segmentation of objects in the image. Due to the high demand for fine-grained segmentation in semantic segmentation models, the Conv_Mask module does not employ shared convolutions to maintain segmentation accuracy. Furthermore, to reduce parameters and computational load, the improved segment layer replaces one 3 × 3 convolution kernel with a 1 × 1 convolution kernel, optimizing model efficiency while preserving performance.

**Figure 9:**
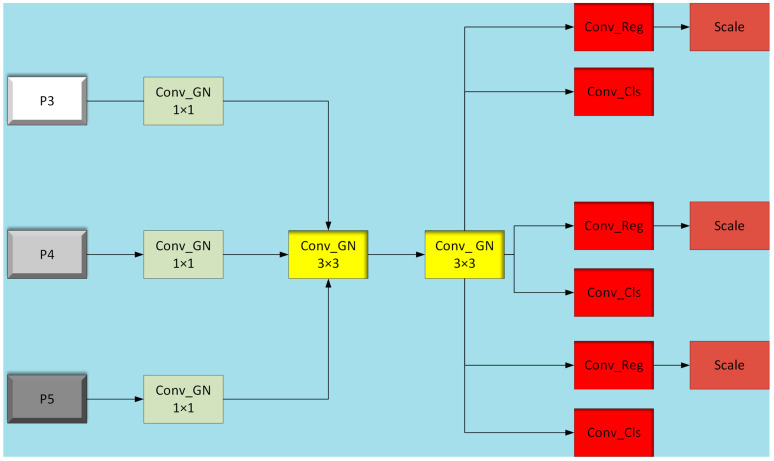
Head layer structure with introduced shared convolutions.

The original design of the YOLOv8 head layer is depicted in Figure 7, where features from three different scales, P3, P4, and P5, are processed independently through respective branches. Each branch consists of two 3 × 3 convolutional layers and one 1 × 1 convolutional layer. While this design enhances detection accuracy, it simultaneously results in a significant increase in the number of convolutional layer parameters. To address this issue, this paper introduces the concept of shared convolution within the detection head, as illustrated in Figure 9. Specifically, we redesign the two 3 × 3 convolutional layers originally distributed across multiple branches, as well as the convolutional layers for classification and regression, to be shared. This sharing mechanism means that the three feature branches at different scales will utilize the same set of weight parameters during convolutional processing. Such sharing significantly reduces the number of model parameters, lowers model complexity, and potentially enhances computational efficiency. Furthermore, by sharing convolutional layers, both classification and regression tasks extract information from the same input features, ensuring consistency between the two tasks. This consistency contributes to improving the model’s performance in classification and regression tasks. Moreover, since classification and regression tasks share computational resources, the model can more efficiently utilize computing power during training, speeding up the training process and reducing training time. Importantly, through shared convolutional layers, the model learns feature representations across tasks, thereby enhancing its generalization ability. This capability allows the model to classify and regress better when facing new, unseen data. Additionally, reducing the number of parameters and computational complexity makes the model more lightweight, suitable for deployment on resource-constrained devices, thereby providing greater flexibility for practical deployment.

In summary, this paper proposes a strategy of sharing convolutions for classification and regression operations within the segment layer, aiming to optimize the model’s parameter efficiency and computational performance while maintaining or improving its performance in classification and regression tasks. This design not only enhances the model’s generalization ability, but also makes it more applicable to real-world application scenarios.

#### 3.4.5. Group Normalization

During the training process of neural networks, the iterative update of model parameters is a critical step where changes in current layer parameters are often influenced by preceding layer parameter changes. This hierarchical propagation characteristic can lead to the problem of gradient vanishing in shallow neural networks during backpropagation, thereby hindering the network’s convergence performance. To effectively address the challenge of gradient vanishing, batch normalization techniques are commonly employed in network architecture design [27]. Batch normalization transforms input data to approximate a standard normal distribution, enhancing the responsiveness of nonlinear functions to input data. This method accelerates the network’s convergence speed and improves prediction accuracy.

However, the effectiveness of batch normalization is significantly affected by batch size. Particularly in small batch scenarios, applying batch normalization may increase errors in output results, thereby affecting model performance. Therefore, careful selection of batch size is crucial to ensure that batch normalization achieves optimal effectiveness. To overcome the impact of batch size on normalization effectiveness, this paper proposes an innovative approach: replacing the original batch normalization in the LSCD convolutional layers with group normalization (GN) [28]. GN is a more robust normalization method that maintains stable performance across batches of different sizes. By adopting GN, we can mitigate the undue influence of batch size selection on normalization effectiveness, ensuring that the model performs well in various scenarios. This improvement not only enhances the convergence speed and prediction accuracy of the model, but also strengthens its robustness and generalization capability.

**Figure 10:**
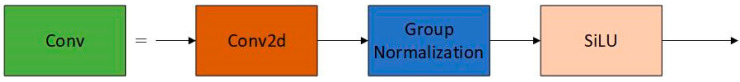
GN structure diagram.

Group normalization (GN) is a normalization technique used in convolutional neural networks. Unlike BN, GN operates by grouping channels and normalizing within each group. This approach reduces the impact of batch size on model performance. GN’s computation is independent of batch size, making it stable even for small batch sizes used in high-precision image scenarios. Additionally, GN computes mean and variance within each channel group, which helps reduce noise and improves model stability. The formula for group normalization (GN) is as follows:

(14) $$GN(x)=\frac{x-u}{\sqrt{\theta^{2}+\varepsilon }}$$

where x is the input feature, μ  is the mean computed within each group, $\theta^{2}$ is the variance computed within each group, and  ϵ is a small value. Specifically, if we have a feature x with shape   (N,C,H,W), where N is the batch size, C is the number of channels, and H and W are spatial dimensions, first, the C dimension is divided into G groups, each with C/G channels, and then each group’s features are normalized. According to Reference [29], GN has been shown to improve the performance of detection heads for localization and classification in the FCOS paper. Reference [5] demonstrates that removing group normalization in classification and regression reduces model accuracy by 1%. Therefore, to maintain the detection performance of the detection heads, GN modules are added to the convolutional layers in LSCD, as shown in Figure 10.

#### 3.4.6. Attention

This study utilizes the attention mechanism SegNext_Attention from the SegNeXt semantic segmentation model. SegNeXt, proposed by Guo et al. [30] in 2022, is an innovative network architecture meticulously designed for semantic segmentation tasks, significantly enhancing performance. This architecture disrupts traditional convolutional attention design principles by introducing the multi-scale convolutional attention module (SegNext_Attention), which effectively encodes contextual information. Compared to traditional self-attention mechanisms in transformers, SegNext_Attention demonstrates higher performance.

At the core of SegNeXt lies the SegNext_Attention module, where the attention mechanism is cleverly integrated between the encoder and decoder connections. This mechanism learns pixel-level attention weights, allowing the model to precisely focus on regions of interest while disregarding irrelevant background information. The overall framework of SegNext_Attention is carefully constructed, comprising encoder, attention mechanism, decoder, and loss function components.

The encoder consists of multiple convolutional and pooling layers, progressively reducing feature map size and increasing channel numbers for efficient feature extraction. On the output feature map of the encoder, the SegNext_Attention mechanism generates attention weights. The decoder utilizes up-sampling and convolution operations to map encoder feature maps to pixel-level segmentation results, gradually restoring feature map size and reducing channel numbers. Ultimately, the training process guides the model through the computation of loss functions, such as cross-entropy and Dice loss, which effectively measure prediction accuracy.

**Figure 11:**
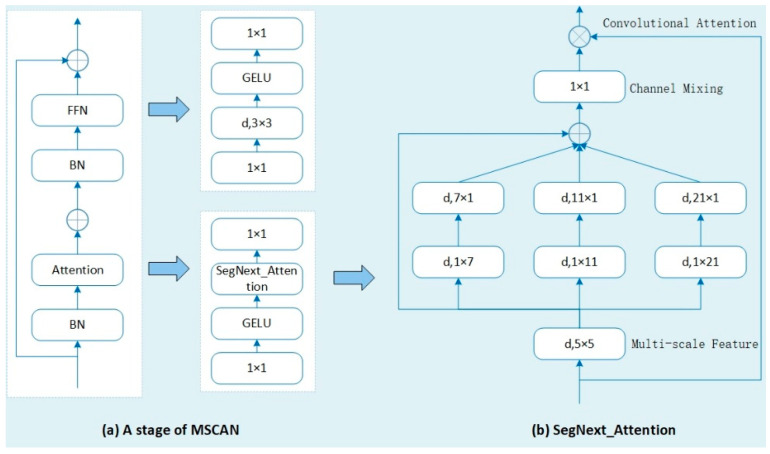
MSCAN and SegNext_Attention structures.

In the encoder section, traditional convolutional block designs are overturned by introducing the multi-scale convolutional attention mechanism. This mechanism leverages multi-scale convolutional features and performs efficient spatial attention computation through element-wise multiplication. The encoder is structured with four down-sampling stages, each employing the same MSCAN module to form a pyramid-like structure for extracting multi-scale contextual feature information, as shown in Figure 11a.

In the decoder section, the Hamburger structure is employed to further extract global contextual information, enabling multi-scale contextual feature extraction from local to global levels. The core design within the MSCAN module is the multi-scale convolutional attention SegNext_Attention, illustrated in Figure 11b. It comprises deep convolution, multi-branch deep convolution, and convolution parts. Deep convolution extracts local feature information, multi-branch deep convolution captures multi-scale contextual feature information, and convolution models correlations between different channels. The output results serve as attention weight parameters, weighting the input to SegNext_Attention and producing the final output. The computation process of SegNext_Attention is depicted in Equations (15) and (16).

(15) $$\;Att=Conv_{1\times 1}\left(\sum_{i=0}^{3}Scale_{i}\left(DW-Conv\left(F\right)\right)\right)$$

(16) Out=Att⊗F

where *F* represents the input feature, *Att* represents the attention weight parameter, *Out* represents the output feature, and ⊗ represents the element-wise multiplication operation. (DW−Conv) denotes the depth-wise convolution operation, $Scale_{i}$ where i∈{0,1,2,3}, represents the *i*-th branch, as shown in Figure 11b. $Scale_{0}$ represents direct connection, while in the other three branches, depth-wise strip convolutions (DWSCs) in two continuous directions are employed to approximate a standard depth-wise convolution with a large core. The kernel sizes for these three strip convolutions are set to 7, 11, and 21, respectively.

The use of vertical and horizontal bar-shaped convolution kernels allows an effective receptive field equivalent to d × d to be achieved while maintaining fewer parameters. Additionally, in the context of separating railway tracks from the ground in transportation systems, these bar-shaped kernels are particularly beneficial for extracting features related to track boundaries. The utilization of depth-wise separable convolution further reduces the parameter count, thereby mitigating the risk of overfitting. The primary advantages of SegNext_Attention include its ability to better utilize global contextual information and focus more accurately on regions of interest, thereby enhancing the performance of image segmentation.

In this study, we propose a train obstacle detection and safety response strategy based on incremental clustering and lightweight shared convolution track segmentation methods. The specific approach is as follows: Firstly, employing incremental clustering algorithms to achieve more accurate clustering results. For each merged cluster, we compute its 3D bounding box. Through joint calibration of cameras and LiDAR, we obtain the transformation matrix T between them. By mapping the obstacle’s bounding boxes to the 2D image plane, combined with track segmentation models, we enhance the safety of train travel. This involves precisely identifying and responding to potential obstacles on the track, ensuring the safety and efficiency of train operations. The formula for mapping 3D obstacle bounding boxes to the 2D image plane is as follows:

(17) $${\mathrm{ProjectedPoints}}_{2D}=\mathrm{Pr}oject(B_{k},T)$$

Using the improved YOLOv8 semantic segmentation model for railway area segmentation in images, we obtain segmentation masks \(M\). The strategy for detecting traffic obstacles is as follows: For each projection point \(x\), check if this point lies within the railway segmentation area \(M\), as shown in Equation (9). If any projection point \(P_{2D}\) falls within the railway area, trigger a safety alert.

(18) $$\mathrm{IsOnTrack}(p_{2D},M)=\{\begin{array}{c} True\;,\\ False\;, \end{array}\begin{array}{c} {\;\mathrm{if}\;M(p}_{2D})=RailTrack\\ \mathrm{otherwise} \end{array}$$

## 4. Results

To validate the effectiveness of the proposed method, we arranged various obstacles such as fallen rocks, toolboxes, and pedestrians in real railway scenarios and collected experimental data on an actual train platform. The experimental platform utilized the Ubuntu 20.04 operating system, with C++ as the programming language. The experimental setup was equipped with a Livox-15 LiDAR, an MV-CS020-10GC camera, and an MVL-KF1624M-25MP lens as detection devices. Data processing was performed on an industrial computer with a Core I9-11900K CPU, an RTX-4060 GPU, and 16 GB of RAM. The LiDAR and camera were used to capture the environment in front of the train, with the image and 3D point cloud data being sent to the industrial computer for computation. An improved semantic segmentation algorithm was utilized to obtain the train’s forward driving area, and an incremental clustering algorithm was employed to perceive the obstacle positions ahead of the train. Combined with the train’s safe driving strategy algorithm, this setup enhances the operational safety of the train.

Based on this setup, we conducted two experiments to further verify the effectiveness and practicality of the proposed detection strategy. First, we performed a lightweight shared convolutional rail segmentation experiment, focusing on optimizing the algorithm’s accuracy in recognizing and classifying tracks in complex scenarios. This step aimed to enhance the system’s ability to process images, particularly in determining the position of obstacles during the train’s safe operation, through improvements to the YOLOv8 algorithm. Additionally, the improved algorithm was validated through ablation and comparative experiments, which not only demonstrated the performance differences before and after the algorithm’s improvement, but also compared our method with current state-of-the-art technologies. The proposed algorithm demonstrated high accuracy in track recognition and real-time detection, showcasing its potential for practical applications in railway traffic safety.

### 4.1. Experimental Environment

In the training phase of this study, the deep learning framework was deployed on the Ubuntu 20.04 operating system with an Intel Xeon Silver 4210R CPU, operating at a clock speed of 2.4 GHz with 40 cores, and an NVIDIA GeForce RTX 4090 GPU with 24 GB of memory. Python version 3.8 was used, along with PyTorch version 2.2.1 and CUDA version 12.1. The experiment utilized the Cityscapes+Railsem19 railway dataset for both training and testing the network. This dataset is specifically designed for urban scene segmentation and visual detection in railway scenarios, encompassing various railway-specific elements suitable for evaluating and optimizing deep learning algorithms.

### 4.2. Figures, Tables, and Schemes

To evaluate the effectiveness of the model for semantic segmentation in railway traffic, several evaluation metrics were utilized, including mean average precision (mAP), frames per second (FPS), computational load (GFLOPs), and parameter count (M/params). These metrics collectively assess the model’s performance in accurately segmenting railway scenes, considering both precision and efficiency aspects crucial for real-world applications in railway traffic safety.

1. Precision (P): Represents the proportion of correctly predicted samples among all samples predicted as positive by the model, as shown in Equation (19).

(19) $$P=\frac{TP}{TP+FP}$$

2. Recall (R): Represents the measure of coverage, indicating how many positives were predicted correctly. It measures how well the algorithm’s model can identify more defects in the image, calculated as shown in Equation (20).

(20) $$R=\frac{TP}{TP+FN}$$

3. Average precision (AP): Based on precision and recall, AP averages the precision values, calculated as shown in Equation (21).

(21) $$AP=\int_{0}^{1}P(r)\mathrm{dr}$$

4. The formula for calculating mask average precision is shown as Equation (22).

(22) $$mAP_{mask}=\frac{\sum_{0}^{N}\int_{0}^{1}p_{n}(r)d_{r}}{N}$$

In the above formula, N represents the number of classes/categories in the dataset, Pn denotes the average precision (AP) for a specific class. mAP@0.5:0.95 refers to the mean average precision (mAP) averaged over multiple AP scores calculated at IoU thresholds ranging from 0.5 to 0.95 with a step size of 0.05. mAPmask@0.5 specifically indicates the average precision mean at an IoU threshold of 0.5.

### 4.3. Ablation Experiment

**Figure 12:**
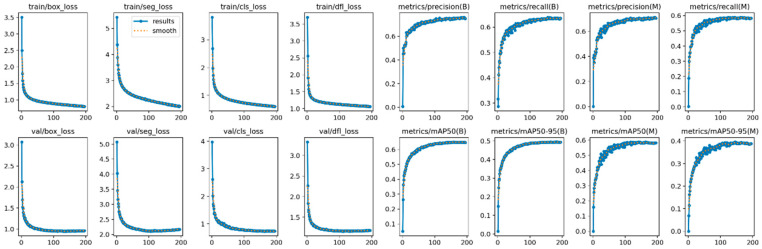
Loss function and performance metrics.

The loss function serves as a critical metric to assess model performance, describing the disparity between predicted values and ground truth. To evaluate the impact of improvements made to various modules in this study on the overall model performance, visualizations of the loss function and performance metrics of YOLOv8s-seg after enhancements were conducted, as depicted in Figure 12. From the graph, it is evident that the training has converged effectively.

**Figure 13:**
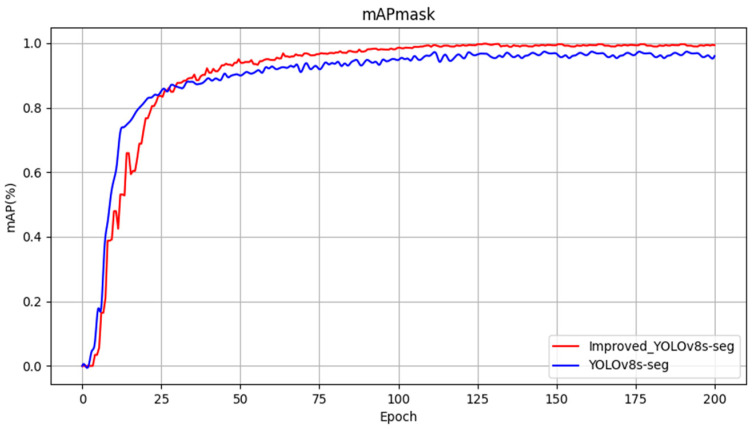
Comparison curve of mAPmask before and after improvement.

**Figure 14:**
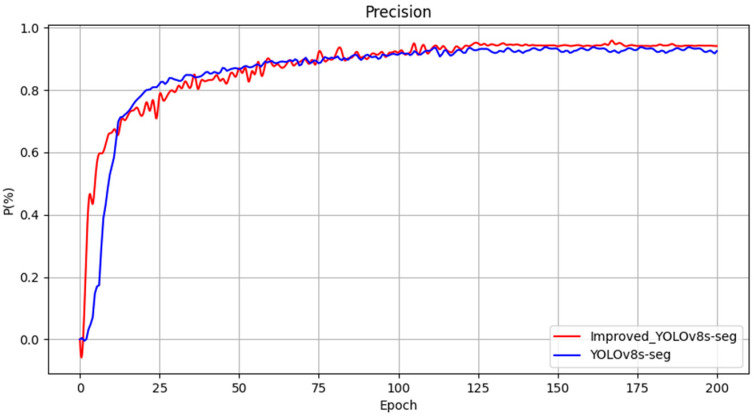
Comparison curve of precision before and after improvement.

**Figure 15:**
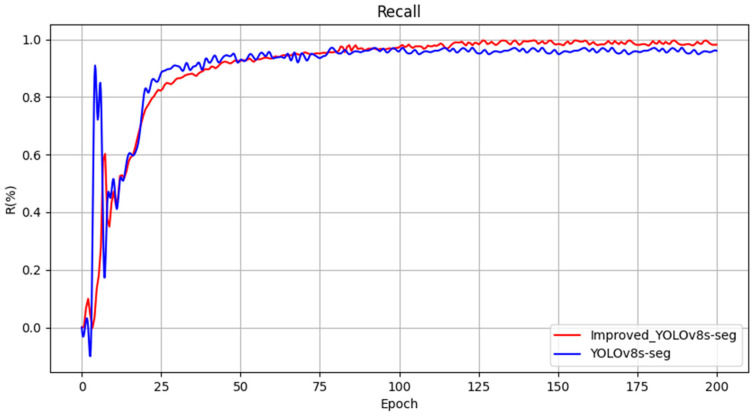
Comparison curve of recall before and after improvement.

The evaluation metrics chosen for assessing the detection performance of the models include mAPmask (mean average precision with masks), precision (P), and frames per second (FPS). Four experimental groups were designed and their training results are presented in Table 2. According to Table 2, several observations can be made. Initially, Model 1 achieved an mAPmask of 97.3% under the original YOLOv8s-seg model configuration. Model 2, incorporating SegNext_Attention into the backbone network, showed improvements with a 1.5% increase in mAPmask, 0.7% increase in precision, and 1.5% increase in recall compared to Model 1. Model 3, which introduced a custom lightweight shared convolutional detection head, further enhanced mAPmask by 1.8%, Precision by 1%, and recall by 2.3% relative to Model 2. Lastly, Model 4, the proposed enhancement method presented in Figure 13, Figure 14 and Figure 15, demonstrated significant improvement in detection performance compared to the original model. Specifically, it achieved an increase of 1.8% in mAPmask, 2.1% in precision, and 2.5% in recall. Furthermore, the improved model exhibited a 58% reduction in computational load and achieved an FPS of 121.9, thereby meeting real-time performance requirements while ensuring accuracy. These findings highlight the effectiveness of the proposed improvements in enhancing both the accuracy and efficiency of the model for semantic segmentation tasks in railway traffic scenarios.

### 4.4. Comparative Analysis of Performance Results across Different Algorithms

To validate the advantages of the proposed improvement algorithm, this study conducted comparative experiments between the existing mainstream YOLO series semantic segmentation algorithms and the proposed LSCD_YOLOv8s-seg model. Comprehensive evaluations were conducted based on performance metrics including precision, segmentation mean average precision (mAPmask), model size (size), floating point operations (FLOPs), recall, and frames per second (FPS). Specifically, as shown in Table 3, YOLOv5-6.0-seg, YOLOv7-seg, and YOLOv8s-seg were selected as comparative models. Experimental results demonstrate that the enhanced LSCD_YOLOv8s-seg model exhibits significant improvements across all performance metrics. Particularly noteworthy are its advancements in precision, recall, and mAPmask, while maintaining lower computational burden and higher real-time performance. Compared to other models, the improved YOLOv8s-seg demonstrates an exceptional balance between performance and efficiency, offering a superior solution for semantic segmentation tasks in railway traffic scenarios.

The improved model proposed in this study shows notable enhancements compared to other algorithms, as indicated in the table. Specifically, mAPmask has been improved by 6.6%, 4%, and 2.5% respectively, Precision has increased by 5%, 2.8%, and 2.1% respectively, and recall has risen by 3.3%, 1.7%, and 2.5% respectively. Moreover, the improved model achieves significant optimization in terms of model size and computational load compared to the original model. Additionally, it achieves an FPS of 121.9, meeting real-time requirements effectively.

### 4.5. Experiment on Obstacle Intrusion Boundary Detection

Using the improved YOLOv8 railway segmentation algorithm, we conducted experiments on barrier intrusion boundary detection to test its ability to accurately identify and locate obstacles within the track boundaries during train operation. This series of experiments aimed to ensure the practical application effectiveness of the detection strategy in real railway environments, thereby enhancing train operational safety. In this study, as illustrated in Figure 14, to comprehensively evaluate the performance of the proposed approach in real-world application environments, we collected a dataset comprising 10,300 frames of point cloud data, representing a comprehensive railway scene. The study simulated various common obstacles on the railway tracks, including sudden falling rocks, toolboxes left by workers, and pedestrians, among others, to simulate multiple emergency scenarios that may occur during railway operations. Many of these obstacles may not have been learned by the target detection algorithm, highlighting the critical importance of the algorithm’s capability in ensuring safe train operation even in the face of unforeseen obstacles.

**Figure 16:**
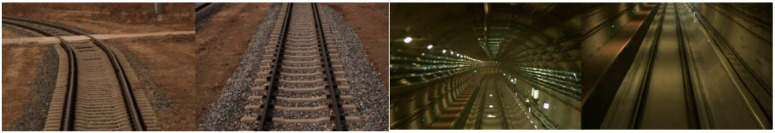
Real-world operational scenarios.

To thoroughly assess the robustness of the system, comprehensive evaluations were conducted under stringent test conditions. This research aims to ensure that the proposed solution maintains high efficiency and accuracy when confronted with various challenges in railway environments. The dataset included 12,600 frames of obstacle-free data and 8700 frames containing obstacles, all located within the LiDAR range of 0–300 m. Specifically, the dataset also included collections of various obstacles such as cardboard boxes, pedestrians, falling rocks, and other railway-related elements, comprising 2600 frames, 2720 frames, 3600 frames, and 3680 frames, respectively. Through this experimental design, as shown in Figure 16, our study comprehensively evaluated the application effectiveness and robustness of the proposed solution in real railway scenarios.

#### 4.5.1. Qualitative Analysis

This study aims to evaluate the application performance of the proposed 3D LiDAR detection system in railway traffic environments. Through the careful design of five representative experimental scenarios, this research examines the system’s accuracy and robustness in identifying obstacles under different environmental conditions. Each scenario is subjected to dual experimental settings: one where obstacles encroach upon the track boundary and another where obstacles do not, thereby comprehensively assessing the system’s false alarm and miss rates. To further validate the safety assurance of integrating the incremental clustering algorithm and lightweight shared convolution algorithm during train operation, curved track experimental scenarios were specifically included. The inclusion of curved track experiments is motivated by the unique visual changes and spatial layouts inherent to curved environments in railway traffic, posing greater challenges to obstacle detection systems. The improved YOLOv8 semantic segmentation algorithm efficiently processes visual information in curved sections, enabling accurate identification of track boundaries. Demonstrating high robustness and adaptability in complex railway traffic environments, this study showcases the algorithm proposed herein.

**Figure 17:**
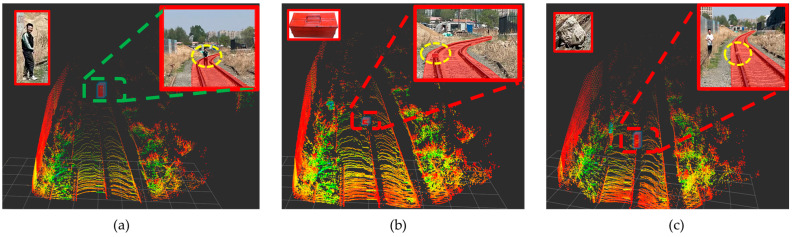
Representation images of experiments on intrusion limits of obstacles, where (**a**) depicts pedestrian detection, (**b**) depicts toolbox detection, and (**c**) depicts foreign object detection.

As shown in Figure 17, Experiment A focused on testing scenarios where obstacles intrude onto the railway track. Various obstacles, such as heterogeneous objects, toolboxes, and pedestrians, were placed on the track to evaluate the system’s detection rate and accuracy. The detection results depicted in the figure demonstrate that the system accurately identifies and locates all obstacles, confirming its efficient performance and low false positive rate in complex backgrounds.

**Figure 18:**
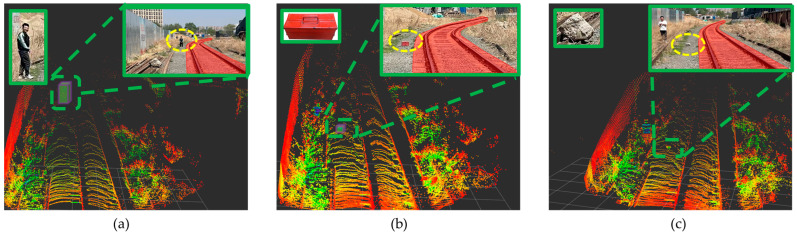
Experimental demonstration images outside the obstacle boundary, where (**a**) depicts pedestrian detection, (**b**) depicts toolbox detection, and (**c**) depicts foreign object detection.

Experiment as shown in Figure 18 (obstacle does not intrude onto the track): The obstacle is placed near the curve but does not intrude into the track area. The system accurately identifies the obstacle’s position without any false alarms, demonstrating the environmental adaptability and high accuracy of the algorithm proposed in this paper.

After conducting comprehensive qualitative analysis, the proposed train safety detection system, integrating incremental clustering and lightweight shared convolution track segmentation algorithms, has demonstrated significant accuracy and robustness across various complex rail transportation scenarios. The algorithm effectively identifies and precisely locates obstacles in all experimental scenarios. It not only accurately detects obstacles in each scenario, but also shows an average error in obstacle position of less than 10 cm compared to actual measured data, meeting the operational requirements for train applications in real-world scenarios.

These achievements not only validate the practicality of the system in enhancing railway safety and efficiency, but also provide important scientific support and practical references for obstacle detection technologies in railway environments.

#### 4.5.2. Quantitative Analysis

To further validate the accuracy of the proposed method, parameters such as false alarm rate and miss detection rate are used to evaluate the detection results of obstacles, as formulated in Equations (23) and (24).

(23) $$P=\frac{M}{M+N}\times 100\%$$

In the equation, P represents the false alarm rate; M denotes the number of false alarms, measured in occurrences; and N signifies the total number of correct detections, measured in occurrences.

(24) $$S=\frac{Q}{Q+N}\times 100\%$$

In the equation, S represents the miss detection rate; Q denotes the number of missed detections in units of occurrences; and N represents the total occurrences, which is the sum of M and Q. From these definitions, it is evident that lower false alarm rate $P_{F}$ and miss detection rate $P_{M}$ correspond to better performance.

In this study, to validate the effectiveness and superiority of the proposed obstacle detection method in real-world environments, we systematically compared it with several existing advanced methods. These comparative methods include a laser radar-based obstacle detection method [22], an improved noise-aware Density-Based Spatial Clustering of Applications with Noise (DBSCAN) algorithm [31], and an enhanced version of the R-CNN model [9]. Each of these methods demonstrates specific advantages in their respective domains: the laser radar method excels in processing three-dimensional spatial information, DBSCAN shows significant improvements in long-range obstacle recognition, and the R-CNN model performs well in terms of recognition speed and accuracy. Through comparisons with these methods, our aim is to demonstrate the comprehensive performance of the proposed method in terms of obstacle detection accuracy, speed, and adaptability, thereby proving its application potential in complex railway or industrial park environments. Comparative experiments on obstacle detection across 12,600 continuous frames of three-dimensional point cloud data are detailed in Table 4 to illustrate the detection outcomes.

In this study, we analyzed and compared the performance of three obstacle detection methods: Method 1, Method 2, and the proposed algorithm in this paper. The proposed algorithm achieved a 4.58% increase in pedestrian detection rate compared to Method 1, reaching 98.2%, demonstrating superior detection effectiveness. Furthermore, for toolbox detection, the proposed algorithm also exhibited higher accuracy, improving by 3.5% compared to Method 1. In detecting heterogeneous obstacles, the proposed algorithm achieved a detection rate of 90.3%, which is 6.1% higher than Method 1, highlighting significant advantages. Method 2 performed poorly in detecting heterogeneous obstacles with a detection rate of 0%, attributed to its supervised learning approach, which fails to effectively recognize previously unlearned obstacle types. Moreover, Method 2 showed lower detection rates for pedestrians and toolboxes, particularly dropping significantly beyond distances of 100 m. Specifically, Method 2 achieved a pedestrian detection rate of only 92.0% and a toolbox detection rate of 60.3%. The proposed algorithm not only significantly improved detection distances, but also demonstrated higher accuracy and reliability in detecting pedestrians, toolboxes, and heterogeneous obstacles. Compared to Methods 1 and 2, the proposed algorithm exhibits clear advantages in handling longer distances and identifying previously unlearned obstacle types, underscoring its broad application potential.

## 5. Conclusions

This study addresses the problem of obstacle detection in railway transportation by proposing a method that integrates an incremental clustering algorithm with lightweight shared convolutional railway segmentation technology for safe train operation. This approach resolves challenges faced by traditional deep learning object detection techniques in handling unknown or sudden obstacles, effectively identifying any unexpected obstacles within the track boundaries, significantly enhancing the overall performance of the detection system, and meeting the high safety requirements of train operations. In terms of object detection, the study employs an incremental clustering algorithm as the backbone for detection, capable of accurately detecting potential physical objects or obstacles. This method is particularly suitable for detecting random and unknown obstacles in railway traffic environments. Furthermore, the research effectively integrates the incremental clustering algorithm with railway segmentation methods to achieve precise obstacle localization.

Building upon YOLOv8-seg, the study introduces a custom lightweight shared convolutional detection head and an adaptive feature selection module to capture global spatial information from railway images for regional segmentation, thereby further improving the accuracy and reliability of obstacle recognition. This comprehensive strategy not only significantly enhances the real-time and accuracy aspects of obstacle detection in front of trains, but also makes a significant contribution to enhancing train operation safety. The three-dimensional point cloud data processing method proposed in this study surpasses traditional techniques in efficiency and accuracy. The average processing time of traditional methods is reduced from 0.1271 s to 0.0971 s, representing a reduction of approximately 23.6%. The detection rate for unknown or sudden obstacles exceeds 90.3%, demonstrating the innovative contributions of this research to urban railway transportation safety. The application of these technologies not only improves the operational efficiency and safety of public transportation systems, but also provides transferable technological solutions for other relevant fields. Overall, this study demonstrates outstanding performance in the field of obstacle detection technology in railway transportation, making a significant contribution to enhancing the safety and reliability of urban railway transportation systems.

## 6. Limitations

The proposed method exhibits certain limitations when applied to complex external environments. One major limitation is the sensitivity of the system to varying light conditions, which can significantly affect the performance of the camera sensors. In environments with strong or weak light sources, the interference can lead to decreased detection accuracy and reliability. Moreover, the proposed method is currently designed for specific railway environments, and adapting the algorithm to handle a wider range of environmental conditions requires further research. Incorporating additional sensor modalities, such as radar and thermal imaging, could enhance the robustness and precision of the detection system, but this would also increase the complexity and computational requirements of the system.

Future research should focus on optimizing the algorithm to better handle varying lighting conditions and long-distance detection challenges. Exploring the integration of multiple sensor data will be crucial in improving the overall robustness and accuracy of obstacle detection in diverse and complex railway environments.

## Figures and Tables

**Table 1:** Performance metrics of the five YOLOv8-seg models.

Model	Size (Pixels)	mAP^box^ 50–95	mAP^mask^ 50–95	Speed CPU ONNX (ms)	Speed A100 TensorRT (ms)	Params	FLOPs
YOLOv8n-seg	640	36.7	30.5	96.1	1.21	3.4	12.6
YOLOv8s-seg	640	44.6	36.8	155.7	1.41	11.8	42.6
YOLOv8m-seg	640	49.9	40.8	317.0	2.18	27.3	110.2
YOLOv8l-seg	640	52.3	42.6	572.4	2.79	46.0	220.5
YOLOv8x-seg	640	53.4	43.4	712.1	4.02	71.8	344.1

**Table 2:** Comparison of ablation experiment results.

SegNext_Attention	LSCD	Recall/%	Precision/%	mAP_mask_/%	Parameters	FLOPs/G	FPS
		0.971	0.938	0.973	27,228,753	110.0	77.8
√		0.986	0.945	0.988	25,260,529	106.6	128.9
	√	0.994	0.948	0.991	9,856,094	39.1	124.7
√	√	0.996	0.959	0.998	11,144,542	46.2	121.9

**Table 3:** Comparison of various algorithms.

Model	Precision/%	mAP_mask_/%	Size/M	FLOPs/G	Recall/%	FPS/%
YOLOv5-6.0-seg	0.909	0.932	15.2	25.8	0.963	166.6
YOLOv7-seg	0.931	0.958	19.5	36.0	0.979	106.5
YOLOv8s-seg	0.938	0.973	54.8	110.0	0.971	77.8
LSCD_YOLOv8s-seg	0.959	0.998	22.6	46.2	0.996	121.9

**Table 4:** Detection performance comparison table.

Detection Categories: Sudden Rockfall (150 × 150 × 150), Box (Obstacle Size 300 × 300 × 300), Pedestrian (1650 × 40 × 30) Unit: mm					Detection Distance
Method	Class of Obstacle	Detection Rate/%	S/%	P/%	
Method 1 [23]	Pedestrian	93.62	6.38	--	0–100 m
	Toolbox	89.3	10.7	--	
	Opposite obstacle	84.2	15.8	--	
Method 2 [25]	Pedestrian	92.0	7.32	0.68	0–100 m
	Toolbox	60.3	35.5	4.2	
	Opposite obstacle	0	98.2	1.8	
Proposed method	Pedestrian	98.2	1.8	--	0–200 m
	Toolbox	92.8	7.2	--	
	Opposite obstacle	90.3	9.7	--	

## Data Availability

The data that support the findings of this study are available from the corresponding author, upon reasonable request.
